# Preservation of the metaproteome: variability of protein preservation in ancient dental calculus

**DOI:** 10.1080/20548923.2017.1361629

**Published:** 2017-08-11

**Authors:** Meaghan Mackie, Jessica Hendy, Abigail D. Lowe, Alessandra Sperduti, Malin Holst, Matthew J. Collins, Camilla F. Speller

**Affiliations:** ^a^ BioArCh, Department of Archaeology, University of York, York, UK; ^b^ Max Planck Institute for the Science of Human History, Jena, Germany; ^c^ Department of Earth Sciences, Natural History Museum, London, UK; ^d^ Servizio di Bioarcheologia, Museo delle Civiltà, Rome, Italy; ^e^ York Osteoarchaeology Ltd; ^f^ EvoGenomics Section, Natural History Museum of Denmark, University of Copenhagen, Copenhagen, Denmark

**Keywords:** Archaeology, dental calculus, destructive analysis, LC-MS/MS, shotgun proteomics, protein preservation

## Abstract

Proteomic analysis of dental calculus is emerging as a powerful tool for disease and dietary characterisation of archaeological populations. To better understand the variability in protein results from dental calculus, we analysed 21 samples from three Roman-period populations to compare: 1) the quantity of extracted protein; 2) the number of mass spectral queries; and 3) the number of peptide spectral matches and protein identifications. We found little correlation between the quantity of calculus analysed and total protein identifications, as well as no systematic trends between site location and protein preservation. We identified a wide range of individual variability, which may be associated with the mechanisms of calculus formation and/or post-depositional contamination, in addition to taphonomic factors. Our results suggest dental calculus is indeed a stable, long-term reservoir of proteins as previously reported, but further systematic studies are needed to identify mechanisms associated with protein entrapment and survival in dental calculus.

## Statement of significance

Proteins are increasingly being explored in archaeological material following developments in high-throughput, high-resolution tandem mass spectrometry. Sourced from archaeological dental calculus, ancient proteins may be used to identify disease processes, microbiome composition and function, bacterial exposure, and food consumption directly from the oral cavity. However, the extent to which the quantity or quality of this protein information varies between samples is unknown, and this could bias comparative interpretations. Additionally, the quantity of dental calculus sample required for successful biomolecular analysis has not been established. Whilst dental calculus does appear to be a stable and rich source of ancient biomolecules, we found that there is substantial individual variability in the quantity of proteins extracted from dental calculus. Further work is needed to understand the mechanisms behind this variability.

## Data availability

Mass spectrometry data has been deposited to the ProteomeXchange Consortium (Vizcaíno, et al., 2014), via the PRIDE partner repository under accession code PXD001362 (for Driffield Terrace and Oxford Street) and PXD001360 (for Isola Sacra).

## Introduction

Technological developments in high-resolution tandem mass spectrometry have substantially improved our ability to access and identify proteins from archaeological materials. The study of ancient proteins is improving the understanding of taxonomy and phylogeny from extinct fauna (Welker, et al., 2015a), the reconstruction of past ecosystems and their exploitation (Vaiglova, et al., 2014; Welker, et al., 2015b), the composition and trade of material objects (Buckley, et al., 2013; Brandt, et al., 2014; von Holstein, et al., 2014; Bleicher, et al., 2015), cultural heritage practices (Kuckova, et al., 2009; Rao, et al., 2014), individual health (Corthals, et al., 2012; Warinner, et al., 2014a), and ancient diets (Buckley, et al., 2013; Shevchenko, et al., 2014; Stewart, et al., 2014; Yang, et al., 2014; Warinner, et al., 2014b; Xie, et al., 2016). Recently, dental calculus - mineralised plaque (tartar) which accumulates on tooth surfaces during life - has emerged as one of the most promising reservoirs for ancient proteins. The mineralized matrix of dental calculus is of high physical hardness and durability, preserving organic microscopic debris and biomolecules. Frequently found on skeletal material, calculus has been described as “one of the richest known sources of ancient biomolecules in the archaeological record” (Warinner, et al., 2015), preserving molecular evidence of oral bacteria, the human host, as well as consumed foodstuffs, all of which can be directly tied to the individual (Warinner, et al., 2015; Weyrich, et al., 2015). Studies have already recognised the potential of calculus for increasing our understanding of ancient human microbiomes (Preus, et al., 2011; Adler, et al., 2013; Warinner, et al., 2015), dietary practices (Warinner, et al., 2014b), host genetics (Ozga, et al., 2016), and individual disease processes and immune responses (Warinner, et al., 2014a).

Proteomic analysis of dental calculus is a relatively new approach in bioarchaeology. Warinner et al. (2014a) was the first to apply shotgun metaproteomic analysis to archaeological dental calculus. Focusing on four Medieval individuals, they identified an abundance of microbial and host proteins, as well as functional interactions related to host defence. Following this, Warinner et al. (2014b) applied a shotgun approach to 92 archaeological remains, dating from the Bronze Age to the 19^th^ century, recovering ancient proteins from all samples. This large-scale study was focussed primarily on the detection of a single dietary protein (ß-lactoglobulin), and did not explicitly explore issues of protein preservation and individual variability across the sites and time periods.

Given the enormous potential of proteomic analysis of ancient dental calculus, it is necessary to understand the extent to which entrapped ancient proteins may be differentially preserved, and identify potential biases and limitations in this approach. Archaeological materials from all contexts are susceptible to taphonomic processes and biomolecular degradation, which reduces the organic content and results in protein fragmentation and chemical modification. These processes occur as a result of activity from environmental microbial proteases (Child, 1995), as well as chemical and physical action on both organic and inorganic phases (Collins, et al., 2002; Smith, et al., 2003). Whilst the mechanisms of ancient DNA (Briggs, et al., 2007; Briggs, et al., 2010; Sawyer, et al., 2012) and bone protein (collagen and osteocalcin) degradation have been explored (Dobberstein, et al,. 2009; Wilson, et al., 2012; Cleland, et al., 2015), understanding the degradation of other proteins, and whole proteomes, remains limited (Buckley & Wadsworth 2014; Kendall, et al., 2016).

In order to explore protein preservation in archaeological dental calculus, we compared proteomic results from deposits of dental calculus from three contemporaneous Roman-era populations. Proteomic assessments of biomolecular preservation include deamidation, (Welker, et al., 2016; Welker, et al., 2017), total number of identified proteins (Orlando, et al., 2013), and proteome complexity (Wadsworth, et al., 2017), but as yet there are no established proxies or criteria for protein preservation. As such, we explored four measures for overall protein preservation, including: a) the total quantity (μg) of protein preserved in each sample; b) the number of mass spectral (MS) queries produced for each sample; c) the number of peptide spectral matches and d) the number of identified proteins. We also assessed rates of deamidation in human collagens, keratins, and alpha-1-antitrypsin. We further examined the correlation between the amount of starting material of dental calculus and overall protein yield as well as the number of protein identifications in order to investigate to what extent the quantity of calculus analysed influenced the proteomic results.

## Materials and Methods

### Archaeological materials

**Figure 1.**
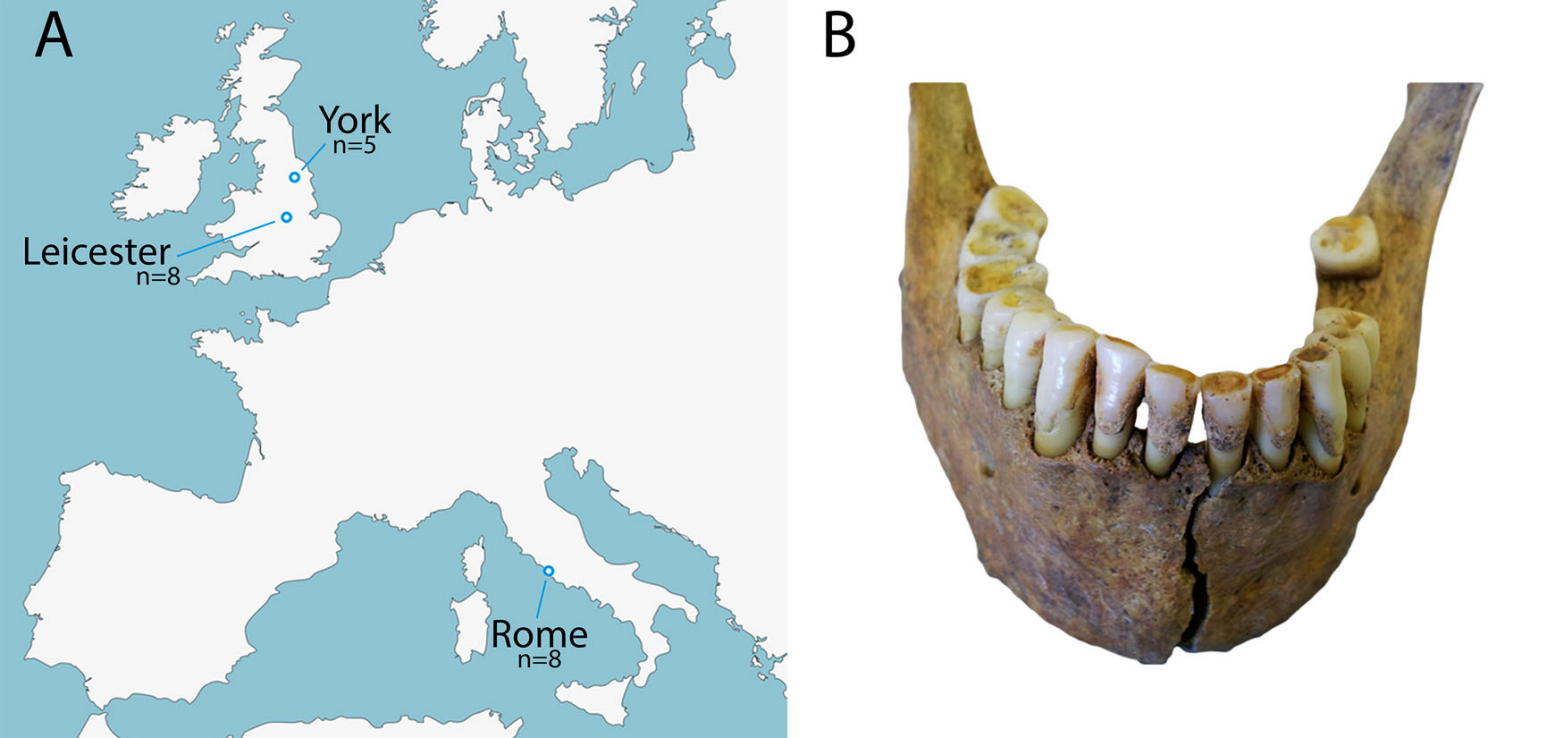
a) Map indicating sample locations of ancient dental calculus used in this study, b) example of dental calculus deposits (individual 3DT26, Driffield Terrace).

Dental calculus was collected from 21 individuals from three sites: two sites located in England (Oxford Street and Driffield Terrace) and one site in Italy (Isola Sacra) (Table 1, Figure 1). Individuals were selected based on the presence of deposits of dental calculus. The burial environments, biological age, sex, and dental pathologies of the sampled individuals are provided in Supplementary Table 1.

**Table 1.** Archaeological dental calculus samples included in this study.

Site	Location	Date	Relative Thermal Age (ka)*	Sample IDs
Oxford Street (Keefe & Holst 2013)	Leicester, UK (NGR SK 586038)	ca. 2nd–5th century CE	1.6	OX01, OX03, OX04, OX05, OX06, OX09, OX10, OX12
Driffield Terrace (Caffell & Holst 2012)	York, UK (NGR: SE 59324510 & SE 59285095)	ca. 2nd–4th century CE	1.2	3DT21, 3DT26, 6DT3, 6DT7, 6DT21
Isola Sacra (Prowse, et al., 2004)	Rome, Italy	ca. 1st–3rd century CE	4.5	SCR227, SCR250, SCR264, SCR323, SCR832, SCR5028, SCR5042, SCR5070

*Illustrative relative differences in thermal age (in thousands of years) assuming an Ea of 127kJ mol^−1^, and equivalent burial depths and soil conditions for all three sites.

### Extraction

Dental calculus was removed from teeth using a sterile dental pick, and calculus deposits from several teeth from the same individual were pooled together for analysis. Calculus deposits were weighed, and between 14–60 mg of calculus was included in the protein extraction (Supplementary Table 1). Proteins were extracted at BioArCh (University of York) using a filter-aided sample preparation method modified for ancient (Cappellini, et al., 2014) and mineralised samples (Warinner, et al., 2014a). LC-MS/MS was performed at the Mass Spectrometry Laboratory of the Target Discovery Institute, University of Oxford. Peptides from each sample were resuspended in 20 μL of 0.1% formic acid and 2% acetonitrile buffer (6 μL injected) and run on a Q-Exactive Hybrid Quadrupole-Orbitrap Mass Spectrometer (Thermo Scientific) using the protocol described in Warinner et al. (2014a). Results from the British individuals have already been partially presented by Warinner and colleagues (2014b), and the same MS/MS protocol and settings detailed there were also used for the samples from the Italian site.

### MS/MS Analysis

Raw MS/MS spectra were converted to searchable Mascot generic format using Proteowizard version 3.0.6839 using the 200 most intense peaks in each MS/MS spectrum. MS/MS ion database searching was performed using Mascot (Matrix Science, London, United Kingdom) against all available sequences in UniProtKB and Human Oral Microbiome (Dewhirst, et al., 2010; http://www.homd.org/) databases. Searches were performed against a decoy database to estimate false discovery rates. Peptide tolerance was 10 ppm, MS/MS ion tolerance was 0.07 Da, with a semi-tryptic search with up to two missed cleavages. Based on previous observations of ancient proteome modification (Cappellini, et al., 2012; Warinner, et al., 2014a), carbamidomethylation was set as a fixed modification (due to treatment with chloroacetamide), and acetylation (N-terminus), deamidation of glutamine and asparagine, glutamine to pyroglutamate, methionine oxidation and hydroxylation of proline (the latter a common modification of bone collagen) set as variable modifications. Protein identifications were supported by at least 2 different peptides, and a default significance threshold (p<0.05) in Mascot was applied. Additionally, identified peptides underwent BLASTp searches through the NCBI database (http://blast.ncbi.nlm.nih.gov/Blast.cgi) to validate their identifications, especially in the case of bacterial species identification to rule out conserved peptides between species.

### Data Analysis

Concentrations of extracted protein were determined using a Qubit fluorometer protein assay (Qubit® 2.0 Fluorometer, ThermoFisher Scientific). The number of spectral queries, the number of spectral queries identified and the number of proteins identified were all reported in Mascot. Statistical analyses were conducted using SPSS (v.21, IBM Corporation). We assessed normality of the data using the Shapiro-Wilks test and tested assumption of homogeneity of variances using Levene's test of equality of variances. Where variance was homogenous, we applied one-way ANOVAs, followed by Tukey post hoc tests; where assumption of homogeneity of variances were violated we applied Welch's ANOVA, followed by Games-Howell post hoc tests. After conducting Shapiro-Wilks tests, we assessed pair-wise rates of deamidation in collagens, keratins and alpha-1-antitrypsin using Wilcoxon Signed Ranks tests. We calculated *a priori* and post hoc power analyses (Cohen, 1988) using the program G*Power 3.1 (Faul, et al., 2007). Deamidation rates were calculated for all identified human keratins and collagens, as well as alpha-1-antitrypsin by running the raw files against the Human database of The Global Proteome Machine (Craig, et al., 2004; http://www.thegpm.org) and comparing the relative intensity of peptides found with deamidated residues to those with no deamidation. In order to illustrate the potential impact of temperature we calculated thermal age (www.thermal-age.eu) for each site at 200 CE, assuming identical burial depth and soil conditions (Table 1).

## Results

Proteins were successfully recovered from all 21 of the ancient samples. Summary of protein yield, spectral counts, peptide and protein identifications can be found in Supplementary Table 1.

### Total Quantity of Preserved Protein

**Figure 2.**
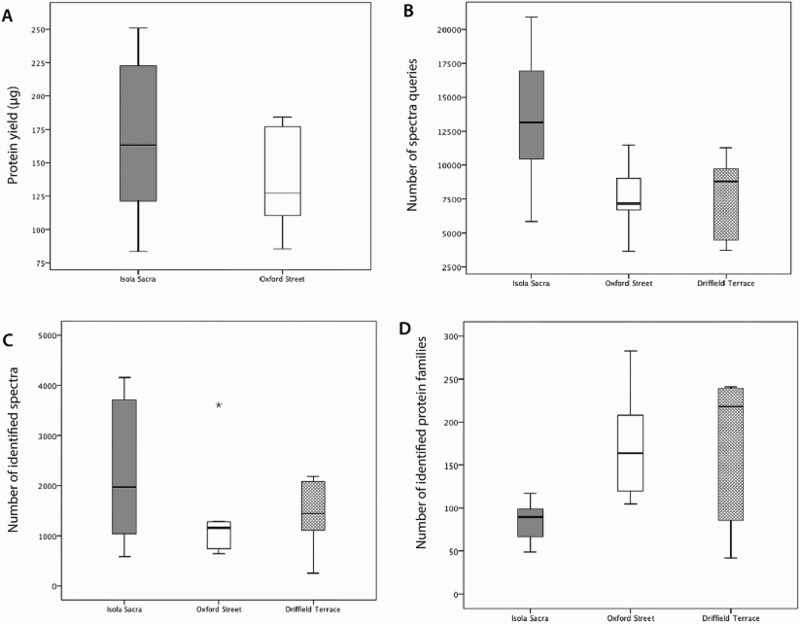
Box plots displaying the range and mean of a) extracted protein yield (μg), b) the number of spectral queries, c) the number of spectral matches and d) the number of identified protein families from the three sites.

We assessed total quantity of preserved protein using a Qubit fluorometer protein assay for the sites of Oxford Street and Isola Sacra (values for the Driffield Terrace site were not available). We examined the yield of extracted protein as a ratio of the starting quantity of material (i.e. yield per milligram of calculus used for analysis). Protein yields from Isola Sacra ranged from 3.24–7.59 μg/mg of calculus (mean=6.02, SD=1.25) while Oxford Street samples ranged from 1.75–9.05 μg/mg of calculus (mean=5.01, SD=2.45) (Figure 2). Comparing the total quantity protein extracted from samples, we find there is no statistically significant difference between the two sites: t(10.442)=1.026, p=0.328.

**Figure 3.**
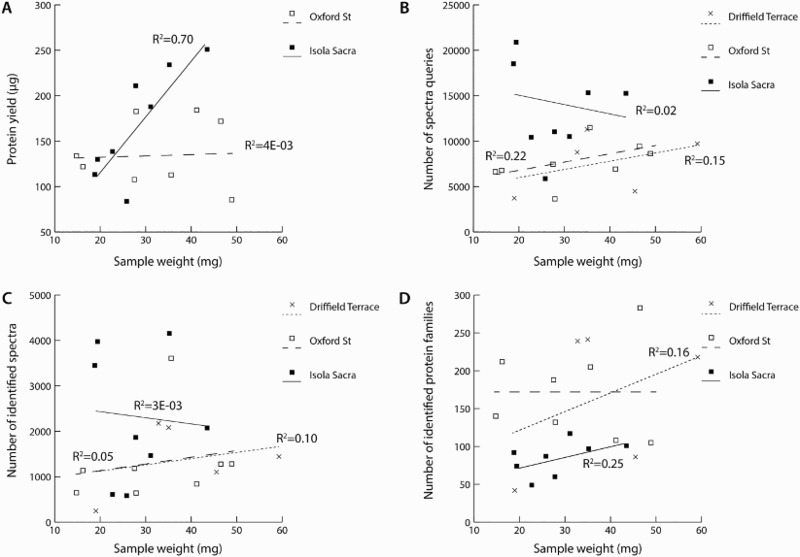
Correlation of quantity of dental calculus sample weight (mg) with a) extracted protein yield (μg), b) the number of spectral queries, c) the number of spectral matches and d) the number of identified protein families.

We assessed the relationship between total protein yield and the starting quantity of calculus using a Pearson's product-moment correlation. Preliminary analyses showed the relationship to be linear with both variables normally distributed, as assessed by Shapiro-Wilk test (p >.05). The Isola Sacra samples displayed a strong positive correlation between the amount of starting material and protein yield (r(6)=.836, p=0.01), but there was no significant correlation between these two variables in the Oxford Street samples (r(6)=.064, p=0.88) (Figure 3).

### Number of Spectral Queries

Next, we assessed the number of spectral queries (MS2, or fragment ions of the whole peptide precursor selected in MS1, used to determine amino acid sequence) produced by LC-MS/MS for Oxford Street, Driffield Terrace and Isola Sacra. We found that the total number of MS2 queries submitted to Mascot range from 3,643–11,488 (mean=7,626.2, SD=2310.9) for Oxford Street, 3,705–11,281 (mean=7,596.2, SD=3324.6) for Driffield Terrace, and 5,872–20,893 (mean=13,478.7, SD=4913.0) for Isola Sacra (Figure 2). When assessed as a ratio of starting material (i.e. normalised by the starting weight of calculus), a one-way ANOVA indicated that although Isola Sacra produced more spectral queries, the different between the sites was not quite significant, Welch's F(2, 11.45)=3.62, p=0.06. The non-normalised query counts, however, were significantly higher for the Isola Sacra groups compared to both British sites, F(2, 18) = 6.117, p=0.009, but no significant differences were observed between the two British sites (p=1.0).

As above, we assessed the correlation between the quantity of starting material and total number of spectral queries (Figure 3). Although the two British sites displayed lines of best fit with an upward trend, none of the sites displayed a significant correlation between the two variables (Oxford Street, r(6)=0.469, p=0.241; Driffield Terrace, r(3)=0.393, p=0.513; Isola Sacra, r(6)=-0.155, p=0.71). Removing the two ‘outliers’ from Isola Sacra (SCR250 and SCR5042), produced a positive line of best fit within the remaining samples, although it too was not significant (r(6)=0.775, p=0.07).

### Number of Identified Spectra

The third analysis compared the number of spectra identified by Mascot in each site (both total number of identified spectra, and as a ratio of the starting quantity of calculus). The total number of identified spectra ranged from 641–3,607 (mean=1327, SD=958) for Oxford Street samples, 250–2,176 (mean=1838, SD=1318.8) for Driffield Terrace, and 581–4,156 (mean=2272, SD=1429) for Isola Sacra (Figure 2). Although Isola Sacra samples produced a higher number of identified spectra overall there were no significant differences between the sites with respect to either normalized (Welch, F(2, 11.145)=1.742, p=0.223) or non-normalized spectral counts (F(2, 18)=1.606, p=0.228). As above, no significant correlations were observed between the starting weight of dental calculus and the number of identified spectra within the archaeological samples (Oxford Street, r(6)=0.235, p=0.576; Driffield Terrace, r(3)=0.309, p=0.613; Isola Sacra, r(6)=-0.058, p=0.89) (Figure 3).

### Number of Identified Proteins

We assessed the number of identified protein families in Mascot, and found that the total number of protein families identified in Mascot ranged from 105–238 (mean=171.6, SD=61.5) for Oxford Street, 42–241 (mean=165.2, SD=94.1) for Driffield Terrace, and 49–117 (mean=84.6 SD=22.4) for Isola Sacra (Figure 2). When assessed as a ratio of starting material, there were no significant differences in protein identifications between the groups (F(2, 18)=3.017, p=0.074). In terms of non-normalised proteins identifications, the Isola Sacra samples displayed significantly fewer protein identifications (Welch, F(2, 7.614)=7.744, p=0.015), although the difference was only significant when compared to the Oxford Street site (p=0.012) and not when compared to Driffield Terrace (p=0.251). No differences were observed between the total protein identifications from the two British sites (p=0.99).

As above, no significant correlations were observed between the starting weight of dental calculus and the number of identified proteins within the archaeological samples (Oxford Street, r(6)=0.003, p=0.994; Driffield Terrace, r(3)=0.339, p=0.506; Isola Sacra, r(6)=0.497, p=0.210) (Figure 3).

### Protein Taxonomic Identifications

Across all 21 samples of dental calculus, we identified a range of host derived and microbial proteins consistent with those identified in Warinner et al. (2014b). Human proteins identified were similar in function to those identified by Warinner et al. (2014b), predominantly associated with the circulatory system, immune response, and regulatory functions. The milk protein ß-lactoglobulin (a dietary protein), was also identified in three Oxford Street individuals and one from Driffield Terrace (Warinner, et al., 2014b): Bacterial proteins belonged to three main groups: commensal, pathogenic and opportunistic pathogenic bacteria. Commensal species are part of the normal microflora of an individual. Pathogenic bacteria cause disease, a subset of which are opportunistic, or those that are normally commensal or non-pathogenic but may cause disease under particular circumstances, such as in immunocompromised individuals. These categories were determined by their inclusion in the HOMD and pathogenicity defined by PATRIC (Pathosystems Resource Integration Center). Commensal species were the most commonly identified group, with Actinomyces the most common taxon. Opportunistic pathogens included *Cardiobacterium valvarum, Corynebacterium matruchotii, Eubacterium yurii, Leptotrichia buccalis,* and *Selenomonas sputigena*, while periodontal pathogens include *Campylobacter rectus, Campylobacter showae, Porphyromonas gingivalis,* and *Tannerella forsythia*.

Any proteins identified in blank extractions, in blank extractions reported in Warinner et al. (2014a), and members of the common Repository of Adventitious Proteins (reported by the Global Proteome Machine, http://www.thegpm.org/crap/) were considered for their likelihood to represent laboratory contaminant (full list in Supplementary Table 2). Contaminant proteins were included in total spectral and protein counts, but reported as contaminants in Supplementary Table 1. Few unique proteins could be assigned to soil bacteria; previous analyses of dental calculus have noted that calculus appears to be more resilient to contamination from soil bacteria than other more porous tissues such as bone and dentine, which are more easily infiltrated by environmental microorganisms (Warinner, et al, 2014a).

**Figure 4.**
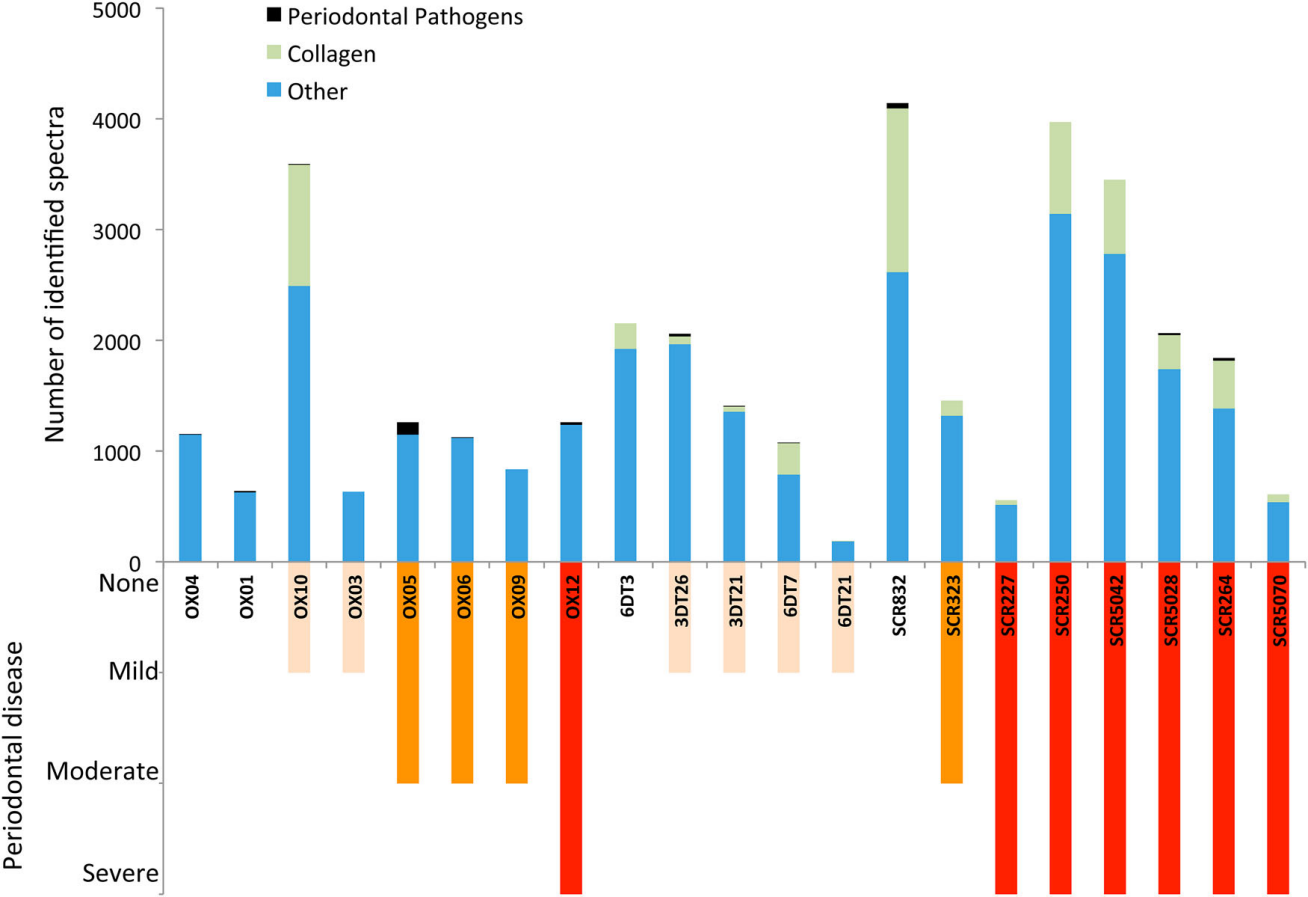
Comparison of number of significant matches assigned to periodontal pathogens and collagen, compared with the severity of periodontal disease as indicated by osteological analysis.

Collagens and keratins were observed frequently. These could result from handling of the samples (e.g. shed human skin cells) as well as resulting from any bone powder incidentally collected during sampling (collagen being the predominant protein in archaeological bone). However, human keratins and collagen may be endogenous to the calculus, incorporated *in vivo* either through their presence in gingival crevicular fluid or potentially as a result of bone degradation during periodontitis. For example, type I and type II keratins have been identified in the gingival crevicular fluid of individuals with chronic periodontitis and periodontally healthy individuals (Bostanci, et al., 2010; Grant, et al., 2010; Baliban, et al., 2012), and are assumed to result from the turnover of oral epithelia. We observed that samples with the highest proportions of significant peptide matches to collagens (OX10, 6DT7, SCR250, SCR264, SCR832, and SCR5042) also included those individuals with the highest number of identified spectra overall (OX10, SCR250, SCR832, and SCR5042, Figure 2, Table 2). Compared to the two British sites, Isola Sacra displayed the highest percentages of collagen peptides. There is little clear association, however, between the prevalence of collagen in the samples and osteological indicators of periodontal disease (Table 2, Figure 4). In order to assess what effect collagens and keratins might have on masking systematic trends in protein preservation between sites, we removed all collagens and keratins from the counts of identified spectra and protein identifications and re-ran the correlation analyses and ANOVAs for these two proxies; no differences, however, were observed in the significance patterns for any of the tests.

**Table 2.** Proportion of significant matches to collagen and keratin identified in dental calculus, compared with osteological indicators of periodontal disease for Oxford Street (Keefe & Holst 2013), Driffield Terrace (Caffell & Holst 2012) and Isola Sacra.

Individual	Total number of proteins	% Collagen (% of identified queries)	% Keratin (% of identified queries)	Periodontal disease (PD)
OX01	140	0.3%	0.6%	No evidence of PD
OX03	132	0.0%	0.3%	Mild PD
OX04	188	0.0%	2.0%	No evidence of PD
OX05	105	0.0%	0.3%	PD
OX06	212	0.0%	1.0%	Moderate PD
OX09	108	0.0%	0.5%	Moderate PD
OX10	205	30.3%	0.4%	Mild PD
OX12	283	0.1%	0.5%	Severe PD
3DT21	218	3.0%	2.6%	Mild PD
3DT26	241	3.6%	0.7%	Mild PD
6DT3	239	10.6%	1.0%	No evidence of PD
6DT7	86	25.4%	3.3%	Mild PD
6DT21	42	3.2%	23.2%	Mild PD
SCR227	87	7.7%	3.8%	Severe PD
SCR250	74	20.9%	0.1%	Severe PD
SCR264	60	23.1%	1.0%	Severe PD
SCR323	117	9.3%	0.9%	Moderate PD
SCR832	97	35.6%	0.4%	No evidence of PD
SCR5028	101	15.0%	0.6%	Severe PD
SCR5042*	92	19.3%	0.1%	Severe PD
SCR5070	49	11.1%	0.2%	Severe PD
Blank Extract 1	60	22.0%	1.8%	
Blank Extract 2	18	29.4%	0.1%	

*Unique match to chicken collagen.

To better understand the source of the collagens and keratins as either modern contaminants or endogenous ancient proteins, we examined levels of deamidation in these two protein groups as well as alpha-1-antitrypsin, a human protease inhibitor found consistently in ancient dental calculus samples (Warinner, et al, 2014a) and unlikely to represent a modern contaminant. The rates of deamidation of asparagine (N) and glutamine (Q) residues to aspartic acid (D) and glutamic acid (E), respectively, have been used as *in vivo* molecular clocks (Robinson & Robinson, 2001; Robinson & Robinson, 2004) as well to estimate the level of degradation in ancient proteins (Wilson, et al., 2012; Schroeter & Cleland, 2016). While asparagine tends to deamidate relatively rapidly, at least in denatured proteins and in flexible regions of native proteins (van Duin & Collins, 1998; Robinson & Robinson, 2001; Robinson & Robinson, 2004), glutamine deamidation is markedly slower and thus can be used to assess protein degradation in archaeological contexts (van Doorn, et al., 2012; Wilson, et al., 2012). Our assessment of bulk deamidation across these three proteins (Supplementary Figure 1, Supplementary Table 1) suggests that on average, 98% of asparagine and 99.5% of glutamine residues in keratins were undamaged. In contrast, deamidation rates in both human collagens and alpha-1-antitrypsin were more advanced, with only 47% and 51% undamaged asparagines and 86% and 54% undamaged glutamines in collagens and alpha-1-antitrypsin, respectively. Wilcoxon signed-rank tests confirmed that both asparagine and glutamine were significantly less likely to be undamaged in collagens and alpha-1-antitrypsin, compared to keratins across individuals (p=<0.002, Supplementary Table 3). Together, the bulk deamidation results suggest that the majority of collagens represent degraded endogenous ancient proteins, while the keratins likely represent relatively recent contamination.

## Discussion

We compared the total yield of extracted protein, the number of submitted and identified MS spectra, and the number of identified proteins between three Roman period sites, finding no systematic trends between populations in any of these measures. Instead, we find substantial variation between individuals, suggesting that these inter-individual differences may outweigh any inter-site variability in this study. While a higher protein yield and a greater number of peptide queries were observed in samples from Isola Sacra than in dental calculus from British sites, this did not translate into a greater number of identified proteins, possibly owing to an abundance of collagen in some of the Isola Sacra samples.

We also correlated the quantity of dental calculus used for analysis with each measure of protein preservation, in order to assess whether using a larger quantity of starting material produces more protein identifications. Virtually no correlation was observed between the quantity of calculus used for analysis and the quantity of preserved proteins. Whilst we did observe a correlation between the amount of starting material and the total yield of extracted protein for samples from Isola Sacra, we note that four of these samples contain a high number of spectral matches that are attributed to collagen. Greater yields of extracted protein, however, did not necessarily equate to a larger number of identified protein families. The results from this study suggest that using larger quantities of dental calculus does not necessarily result in a great number of protein identifications - thus we can be relatively conservative with the amount of material used for analysis. Although it must be acknowledged that we cannot speculate on sample weights under 14 mg, or potentially those in different states of preservation. It is possible, also, that using a greater starting quantity of calculus may ultimately result in improved coverage of selected proteins. Testing this hypothesis, however, is challenging due to significant variation in the lifetime relative abundance of human and microbial proteins in saliva, which are heavily influenced by health status and dietary patterns (Rao, et al., 2009; Gonçalves, et al., 2010; Mandel, et al., 2010; Jou, et al., 2014). The observed persistence of dental calculus biomolecules through time (at least 40 kya, Weyrich, et al., 2017), and our recovery of proteins from different climates, would suggest that biomolecular preservation of calculus is generally robust and that protein recovery may reflect individual variation, as we discuss in further detail below. Given the finite nature of this archaeological material, the destructive nature of the method, as well as the accelerating research in the field of dental calculus, these results have implications for conservators and curators of osteological collections. In this project we obtained over 100 ancient proteins from as little as 15 mg of calculus, although it would not be implausible to yield successful results from even smaller quantities.

### The presence of collagen in dental calculus

Whilst we observed no systematic trends in protein preservation between the three populations, some of the variability between sites and between individuals can be attributed to high quantities of collagen observed in the samples. High protein extraction yields and the highest number of spectral queries were identified from four Isola Sacra samples and one Oxford Street sample; these same samples also had a high percentage of spectral queries matching to human collagenous proteins (in particular, COL1A1 and COL1A2). Collagen should not be a major component of dental calculus, although it is abundant in both cementum and dentine, and has been identified as present in human saliva (Hu, et al., 2005). The deamidation profile of the collagens is more suggestive of an ancient protein, rather than a modern contaminant resulting from human handling. Nevertheless, it is difficult to identify whether the collagen is endogenous to the calculus matrix or results from accidental inclusion of archaeological bone powder during sampling.

To explore whether collagen may be incorporated into dental calculus *in vivo* as a result of periodontitis-associated bone resorption, we explored the relationship between the presence of human collagen and osteological indicators of periodontitis. There was, however, no clear association between the presence of collagen and osteological indicators of periodontal disease (Figure 4). It is most likely that collagen from associated bone powder was incidentally incorporated during dental calculus sampling; this is particularly likely when the maxillary or mandibular bone is friable. As collagen is the most abundant protein in bone, even small amounts of associated bone powder may result in substantial quantities of collagen co-extracted with the dental calculus proteins. Given that ions with higher intensities are preferentially detected by MS, it is possible that the high abundance of spectra derived from collagen may have masked the detection of other less abundant proteins in some of the archaeological individuals.

Some collagen peptides were observed in both our extraction blanks (and in the extraction blanks monitored by Warinner et al. 2014a), and it may be the case that some of these peptides may be the result of previous handling or laboratory contamination. More research exploring proteins extracted from the calculus of living individuals (with and without evidence of periodontitis) may help us to understand the origin and variability of collagen in dental calculus.

Experiments involving the isolation of collagens and keratins, for example by using SDS-PAGE, may be useful for targeting and analyzing these potential contaminants in greater detail. This approach, however, would only be useful if the collagens and keratins derive from modern contamination, and therefore could be discriminated by their higher molecular weight. For ancient collagens and keratins deriving from bone powder or other sources, exclusion lists may be used during LC-MS/MS to mask the abundance of specific keratin and collagen peptides, allowing the mass spectrometer to acquire more relevant peptides, and improving overall protein identifications (Hodge, et al., 2013).

### Inter-individual variability in ancient dental calculus

Although dental calculus appears to be a robust reservoir of host, bacterial and dietary proteins, this study highlights the current knowledge gap concerning the relative preservation of ancient proteins in dental calculus, and inter-individual variability in terms of protein yield and taxonomic/functional diversity. Whilst here we observe that individual variability seems to outweigh inter-site differences, it is uncertain whether this individual variation reflects *in vivo* processes associated with plaque biofilm formation and calcification, the location of the calculus within the mouth (e.g., incisors or molars) or occurs as a result of degradative processes acting upon dental calculus in the archaeological record. For example, ancient DNA analysis of calculus has demonstrated that temperature related depurination affects the rate of DNA fragmentation (Ziesemer, et al., 2015), with sites from warmer climates displaying differential biomolecular preservation. In a comparison of aDNA and proteins in archaeological bones and teeth, Wadsworth and colleagues (2017) also show that preservation of these biomolecules appear to be most dependent on site temperatures, with independent rates and mechanisms from each other. However, it is still unclear to what extent factors like temperature and time degrade proteins in dental calculus, as well as how variation in burial environments (e.g., soil pH, aridity, etc.) or burial practices (e.g., mummification, crypts, etc.) influences protein preservation. In this study, individuals within each of the two British sites were recovered from similar burial contexts, respectively, though the Italian site displayed greater variation in burial practices, including individuals interred in tombs as well as sand/sandy soil (Supplementary Table 1). Although this study lacks the sample size necessary to differentiate taphonomic factors relating to burial context, future studies, comparing protein preservation across deposits of calculus from the same individual would help control for factors associated with burial environment, and provide a better understanding of individual variation within the dental calculus proteome. Additionally, more systematic studies exploring the quantity and quality of proteins preserved in both modern calculus, as well as a range of archaeological time periods, climatic zones, and burial environments, are required to differentiate and better understand individual variation and preservational biases.

### Inter-individual variability and evidence of periodontal disease

Until we have a clearer understanding of the *in vivo* and post-depositional factors influencing protein yield and diversity, it may be difficult to make meaningful comparisons between the ancient proteomes at an individual level. As an example of the confounding nature of inter-individual variability, we explored proteins associated with periodontal pathogens at the three sites. Proteins from bacteria highly associated with periodontal disease (*C. rectus, C. showae, Filifactor alocis, P. gingivalis, T. forsythia*) were found in 12 of the 21 individuals. Overall, the results indicate that the Oxford Street individuals demonstrated the greatest evidence for periodontal pathogens (159 unique peptides total), while those from Driffield Terrace display the least (35 unique peptides) (Figure 4); this pattern was consistent even when normalised by the total number of identified spectra. Interestingly, at Isola Sacra, only half the individuals had proteomic evidence of periodontal pathogens, even though all but one individuals had osteological evidence for alveolar resorption.

Analysis of modern individuals suggests that salivary and gingival crevicular fluid (GCF) proteomes differ between healthy individuals and those suffering from chronic periodontitis (Grant, et al., 2010; Marsh, et al., 2011; Baliban, et al., 2012), in particular, noting a relative increase in the frequency of specific human immune system proteins (e.g., S100 proteins, immunoglobulins) (Wu, et al., 2009; Gonçalves, et al., 2010; Haigh, et al., 2010). Likewise, genetic analyses of healthy and chronic periodontitis individuals identified significant differences in the microbial communities of subgingival plaque (Liu, et al., 2012; Wang, et al., 2013), including the increased prevalence of periodontal pathogens including the so-called ‘red complex’ of *T. forsythia*, *P. gingivalis* and *T. denticola* (Mineoka, et al., 2008; Colombo, et al., 2009; Teles, et al., 2010) in periodontitis. Our proteomic results from the ancient individuals were not consistent with expectations in microbial profiles based on modern individuals, or with the osteological evidence for oral health. The British samples displayed a higher quantity of periodontal pathogen peptides compared with the Isola Sacra samples even though osteological analysis of the Italian skeletal remains suggest poorer overall dental health, and a higher frequency of severe periodontal disease. However, Driffield Terrace, which had the lowest osteological rates of periodontal disease, also had the lowest number and lowest proportion of periodontal peptides detected.

Identifying meaningful inter-individual or intersite-differences in periodontal disease and/or oral health may be confounded by a number of factors, including small sample size, overall protein preservation, as well as the preferential detection of collagen and keratin, referenced above. For example, SCR250 and SCR5042, have osteological evidence of periodontitis but no pathogenic peptides were detected. These individuals also displayed a higher than average frequency of collagen and keratin peptides, which may suggest that these ‘contaminant’ proteins may mask the presence of lower frequency endogenous oral bacteria. On the other hand, individual SCR5070 also had severe alveolar resorption and no corresponding peptides from periodontal pathogens, but with comparatively low levels of collagen and keratin. Therefore, the presence of ‘contaminant’ proteins may not be the only factor influencing the detection of periodontal pathogens.

Currently, meaningful comparisons in the dental calculus profiles between different cultural areas and time periods are limited until the processes directly affecting variability of proteins present are better understood. Although no systematic trends in protein preservation were found between these three populations, comparisons between more temporally and geographically diverse sites may reveal more systematic trends in protein preservation. Future studies will likely need to apply larger sample sizes in order to detect significant differences in protein preservation. The effect size (i.e., partial eta^2^) for the preservation proxies examined in this study (i.e., number of queries, number of identified spectra and number of identified proteins), range from 0.15 to 0.40, indicating that samples sizes of 30–50 individuals per site may be required in order to have sufficient power to distinguish climatic and/or temporal factors from individual variation.

Additionally, further systematic tests are required to assess calculus proteome variability within different areas of the mouth, as well as variation resulting from the extraction technique itself and from different MS/MS runs (Procopio, et al., 2017). In order to gain a better insight into cultural, dietary, and health status differences between archaeological sites using dental calculus, the experimental, *in vivo* and post-depositional factors influencing protein yield and diversity must be better understood in order to counteract their potential influence that may otherwise be interpreted as cultural or demographic factors.

## Conclusion

In order to explore the variability of protein preservation in samples of ancient dental calculus, we analysed 21 samples of ancient dental calculus from three Roman-period sites in the UK and Italy. We found that there were no systematic trends in protein yields between sites, and that individual variability seemed to outweigh any inter-site trends. Additionally, we explored whether using larger quantities of dental calculus for protein analysis results in higher numbers of identified proteins. For the samples tested (14–60 mg), we find no correlation between the quantity of calculus and the number of proteins identified. Given the successful extraction of proteins from all samples, as well as the identification of endogenous host, bacterial and dietary proteins comparable to previous studies, dental calculus is indeed a stable, long-term reservoir of ancient proteins. However, our results indicate that further systematic studies must be conducted to identify to what extent individual variability is influenced by *in vivo* mechanisms, methods of protein extraction, and post-depositional degradative processes, in order to better interpret findings and gain a better understanding of the factors causing inter-personal differences in the past.
